# Spatio-Temporal Attention Model for Foreground Detection in Cross-Scene Surveillance Videos

**DOI:** 10.3390/s19235142

**Published:** 2019-11-24

**Authors:** Dong Liang, Jiaxing Pan, Han Sun, Huiyu Zhou

**Affiliations:** 1 College of Computer Science and Technology, Nanjing University of Aeronautics and Astronautics, Nanjing 210016, China; panjiaxingzz@gmail.com (J.P.); sunhan@nuaa.edu.cn (H.S.); 2 Department of Informatics, University of Leicester, Leicester LE1 7RH, UK; hz143@leicester.ac.uk

**Keywords:** foreground detection, attention model, optical flow, background modeling

## Abstract

Foreground detection is an important theme in video surveillance. Conventional background modeling approaches build sophisticated temporal statistical model to detect foreground based on low-level features, while modern semantic/instance segmentation approaches generate high-level foreground annotation, but ignore the temporal relevance among consecutive frames. In this paper, we propose a Spatio-Temporal Attention Model (STAM) for cross-scene foreground detection. To fill the semantic gap between low and high level features, appearance and optical flow features are synthesized by attention modules via the feature learning procedure. Experimental results on CDnet 2014 benchmarks validate it and outperformed many state-of-the-art methods in seven evaluation metrics. With the attention modules and optical flow, its F-measure increased 9% and 6% respectively. The model without any tuning showed its cross-scene generalization on Wallflower and PETS datasets. The processing speed was 10.8 fps with the frame size 256 by 256.

## 1. Introduction

Detecting foreground plays an important role in an intelligent surveillance system. It is often integrated with various tasks, such as tracking objects, recognizing their behaviors, and alerting when abnormal events occur. However, object detection suffers from non-stationary scenes in surveillance videos, especially in two potentially serious cases: Illumination variation, such as outdoor sunlight changes and indoor lights turning on/off, physical motion, such as ripples on the water surface, atmospheric disturbance, trees swaying and the motion of indoor artificial objects, which include fans, escalators and auto-doors. If the actual background contains a combination of the factors mentioned above, it becomes even more difficult to perform foreground detection.

In order to eliminate illumination changes and dynamic backgrounds, early studies focus on statistical distributions to build the background model [1,2,3,4]. To cover the variation of illumination change, the background model occupies a large range of intensity, so that the detection would be insensitive. Local features can represent the spatial characters [5,6,7,8,9,10] but cannot adapt to many non-ideal cases, such as texture-less background. In addition, conventional algorithms handle gradual illumination changes by updating the statistical background models progressively as time goes by. In practice, this kind of model update is usually relatively slow to avoid mistakenly integrating foreground elements into the background model, making it difficult to adapt to sudden illumination changes and burst motion. Modern deep learning based semantic or instance segmentation approaches could provide high-level semantic annotation for each frame, but ignore the temporal relevance. On the other hand, the obstacle in introducing a more sophisticated learning technique is that foreground detection is a scene-dependent and pixel-wise processing procedure [11] which requires a relatively lightweight training and detection model to reduce the resources occupancy. Essentially, foreground detection in video is an empirical semantic segmentation problem closely related to appearance, motion, and scene structure, which cannot be well solved by either background modeling or frame-based semantic segmentation. Semantic segmentation of appearance could screen regions such as pedestrians and vehicles in the scene but lacks effective motion cue. Motion regions are likely to be foreground targets, but regions of repetitive motions are often not foreground targets. Inspired by the attention mechanism of the human brain, tentative efforts have been made towards applying attention into deep neural networks [12,13]. The idea is to gather high-level features sequentially and decide where to attend to for feature learning steps, which could be a promising tool for foreground detection.

To bridge the semantic gap between low and high level features for foreground detection, in this paper we propose a Spatio-Temporal Attention Model (STAM) to combine the spatial and temporal information, with which the feature engineering and parameter tuning become unnecessary when handling various video scenes. The static frame and its optical flow feed two independent encoders, then high-level features guide the attention modules to re-weight low-level features to reconstruct the foreground in pixel-level.

The contributions of this work are:

(1) The proposed attention modules. While the conventional encoder-decoder connects the low-level and high-level features blindly without any distinction, the proposed model can be seen as an attention-guided weight-able connection encoder-decoder, to preserve the effective connections and suppress the invalid connection;

(2) Cross-scene foreground segmentation. This utilizes only 5% ground-truth samples in CDnet 2014 [14] to train only one model, and all the testing results on different scenes are given by this model. This model shows satisfying performance in untrained scene segmentation;

(3) The introduction of motion cue. The static frame and its optical flow (motion cue) feed two encoders and reorganized by attention modules to reconstruct the foreground in pixel-level. Compared to the model without motion cue, the proposed model makes significant improvements.

This paper is organized as follows. In Section 1, we discus the related work. Section 2 illustrates the proposed Spatio-Temporal Attention Model in detail. Experiment results and related discussions are given in Section 3, and finally conclusions are presented in Section 4.

## 2. Related Work

**Conventional Background Subtraction**

Since observations of the background in image sequences can be considered as stochastic events, many statistical approaches have been employed [15,16]. The background modeling approaches can be classified into two categories: Independent pixel-wise modeling, which employs the statistical processing of time-domain observations to each pixel and spatial-dependence modeling, which employs principles to exploit spatial-dependence among pixels to build a local or global model.

Most of the earlier background modeling approaches tend to fall into the first category. Wren [17] modeled the observations (YUV) of each pixel as a single Gaussian probability density function. To cope with periodic moving background patterns, the Gaussian mixture model (GMM) [18] was proposed. Elgammal [2] employed kernel density estimation (KDE) as a data-driven modeling method. Since KDE is a non-parametric model, it is closer to the real probability distribution than GMM. Hidden Markov models (HMMs) [19] have also been applied to model the background; topology free HMMs were described and several state splitting criteria were compared in the context of background modeling in [19]. All the above methods adopted a learning rate function for updating the background model online. They share a well-known trade-off problem: With a low learning rate, they can not adapt to sudden changes of illumination, e.g., turning on/off a light, while with a high learning rate, slowly moving objects, or temporarily stopped objects will be detected as background.

The second category uses spatial information to exploit the spatial dependencies of pixels in the background. Oliver [20] employed eigenspace decomposition in which the background was modeled by the eigenvectors corresponding to the largest eigenvalues. Sheikh [8] used the joint representation of image pixels in a local spatial distribution (proximal pixels) and color information to build both background and foreground KDE models competitively in a decision framework. Heikkilä and Pietikäinen [5] used a local binary pattern (LBP) to subtract the background and detect moving objects in real time. [21] modeled appearance changes by incrementally learning a tensor subspace representation by adaptively updating the sample mean and an eigenbasis for each unfolding matrix. In our previous research, we pay attention on co-occurrence pixel-pair background models [22,23,24,25]. The models employed an alignment of supporting pixels for the target pixel which held a stable intensity subtraction in training frames without any restriction of locations. The intensity subtraction of the pixel pairs allowed the background model to tolerate noise and be illumination-invariant.

**CNN Based on Foreground Detection**

A surveillance video can be split into frames and then segmented as foreground and background frame by frame. Instance segmentation approaches based on deep convolutional networks have great potential in this task. The approaches could be roughly divided into two families. One relies on the R-CNN proposals, which is a bottom-up pipeline that the segmentation results are based on the proposals and then labeled by a classifier [26,27]. The other family relies on semantic segmentation results [28,29] where instance segmentation following semantic segmentation by classifying pixels into different instances. A state-of-the-art method Mask-RCNN [30], built upon object detectors [31], also depends on the proposals but features are shared by classes, box predictors, and mask generators, then all results are collected in parallel.

The first approach for background subtraction using CNN was proposed by Brahamand Droogenbroeck [32]. It was generated from a temporal median operation over *N* video frames. Afterwards, a scene-specific CNN was trained with corresponding image patches from the background image, video frames, and ground truth pixels. After extracting a patch around a pixel by feeding the patch through the network and comparing it with the score threshold, the pixel is assigned with either a background or a foreground label. However, the network is scene-specific, i.e., can only process a certain scenery and needs to be retrained for other video scenes. Another approach is DeepBS [33], which utilizes a trained CNN and a spatial-median filter to realize foreground detection across video scenes. This approach is fast running, but as the foreground is detected based on independent frame and the temporal relevance of the neighboring frames is ignored. In Cascade CNN [34], CNN branches processing images in different sizes are cascaded together that helps the cascade CNN to detect foreground objects in multi-scale. Temporal information has not been taken into consideration in this model. A recent study [35] proposed a probabilistic model of the features discovered by stacked denoising autoencoders. The model divides each video frame in patches that are fed to a stacked denoising autoencoder, which is responsible for the extraction of significant features from each image patch. Then, a probabilistic model decides whether the given feature vector describes a patch belonging to the background or the foreground.

**Attention Model**

Evidence from human perception process [36] illustrates the importance of attention mechanism, which uses top information to guide bottom-up feed-forward process. The attention mechanism of the human brain is, at a particular moment, always focused on a part of the scene, while ignoring the other parts. The attention mechanism of human brain could be equivalent to a resource allocation model. Tentative efforts have been made towards applying attention into a deep neural network. Deep Boltzmann Machine (DBM) [12] contains top-down attention by its reconstruction process in the training stage. The attention mechanism has also been widely applied to recurrent neural networks (RNN) and long short term memory (LSTM) to tackle sequential decision tasks [12,13]. Top information is gathered sequentially and decides where to attend for the next feature learning steps. In image classification, top-down attention mechanism has been applied using different methods: Sequential process, region proposal, and control gates. Sequential process [36,37] models image classification as a sequential decision. This formulation allows end-to-end optimization using RNN and LSTM and can capture different kinds of attention in a goal-driven way. Li [38] proposed a pyramid attention model for semantic segmentation that contains a feature pyramid and global attention. The former part merges features in at various scales while the later guides the low-level features making fusion with high-level ones.

## 3. The Proposed Approach

### 3.1. Attention-Guided Weight-Able Connection Encoder-Decoder

High-level features have a larger reception field, contain global context, and are good at scene classification but weak in predicting labels for every pixel in input resolution [38]. While low-level features carry much fine grained information which can help high-level features to reconstruct objects’ details during up-sampling process. U-net is an efficient structure to combine these features [39,40], it propagates information from the down-sampling layers to all corresponding symmetrical up-sampling layers. However, U-net concatenates the encoder and decoder features without any selection, so it cannot determinate whether the features chosen are necessary for foreground segmenting or not. The design of the proposed attention structure is inspired by the recent development of a semantic segmentation model [38], which employs high-level features to re-weight the fine-grained features in channel-wise. The proposed model merges the decoder and encoder features through serious attention processes during the decoder phase. In detail, high-level features provide global information to guide attention modules to select (weight) proper low-level features who make a contribution to binary prediction in an input image in which the encoder features are re-weighted by the decoder layers at a pixel-level and concatenated with the later.

### 3.2. Model Structure

**Figure 1:**
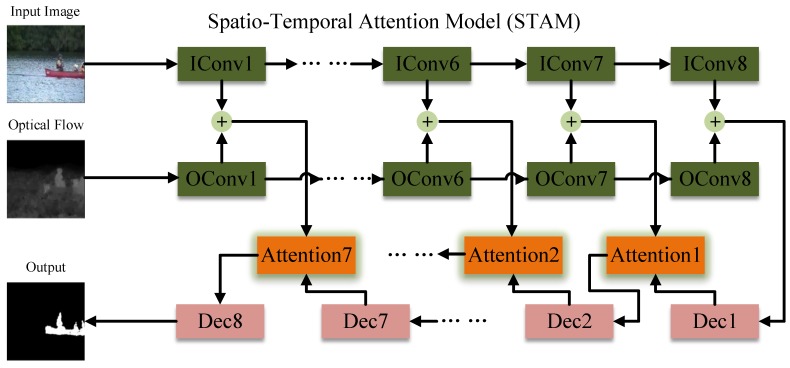
The framework of the proposed foreground detection model, STAM (Spatio-Temporal Attention Model).

As illustrated in Figure 1, the model combines spatial and temporal information, and the attention module is employed to mix encoder features together with decoder ones. The blocks in green represent the encoder layers and “IConv” and “OConv” are two encoders fed with static image and optical flow, respectively. The blocks in pink and orange represent the decoder layers and attention modules. The plus sign in green means the addition in pixel-level while the plus sign in red represents the concatenate operation. For example, there are two feature maps with dimension m×m×n, and there is a m×m×n tensor that goes through addition and a m×m×(2×n) tensor outputted by the later operation.

Table 1 shows details of each layer in STAM. It is fed with a 256×256×3 static image and a 256×256×1 optical flow then outputs a 256×256×1 foreground mask. “IConv” and “OConv” are two encoders with the same structure and eight convolution layers. Additionally, the decoder has eight layers and up-sampling processed in each layer and seven attention modules are applied to make features mixtures. The stride for every convolution is two in both encoder and decoder but one in the attention module. Dropout is utilized to avoid over-fitting in the first three layers of decoder and nodes in these layers with a 50% probability to be dropped in the training phase.

### 3.3. The Proposed Attention Module

**Figure 2:**
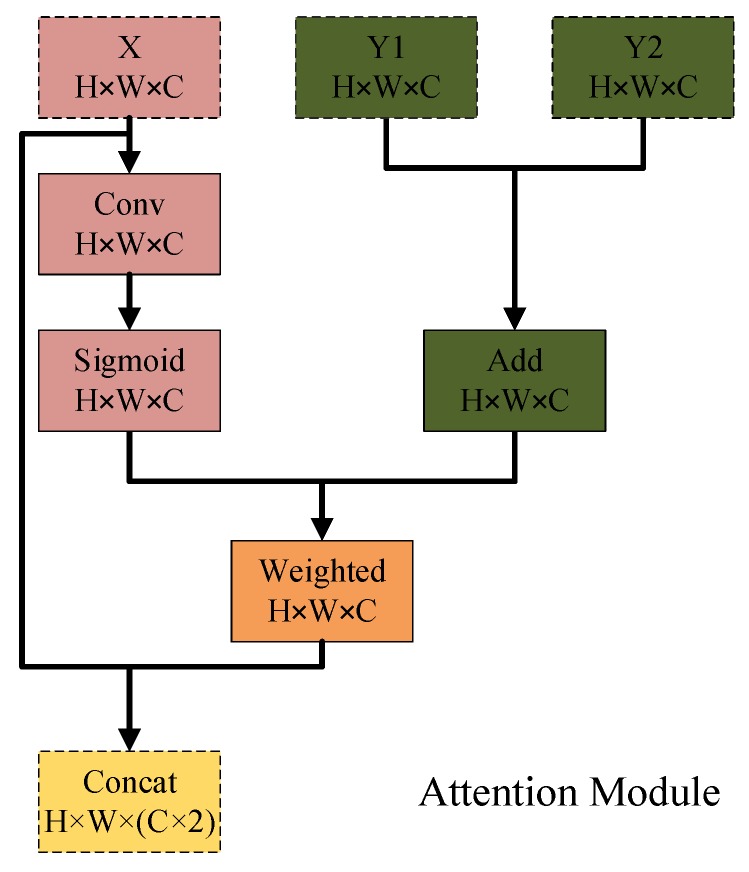
The design of the proposed attention structure.

The design of the proposed attention structure is inspired by a semantic segmentation model [38], employing high-level features to re-weight the fine-grained features in channel-wise. Different from [38], the proposed model merges the decoder and encoder features through a serious attention processes during the decoder phase. In detail, high-level features provide global information to guide attention modules to weight proper low-level features contribute to binary prediction in the inputting image that encoder features are re-weighted by the decoder layers in pixel-level and concatenated with the latter. As shown in Figure 2, the proposed attention modules merge the high-level and low-level features guided by the former ones. Y1 and Y2 are features from image encoder and optical flow encoder, and *X* is the decoder feature respectively. *H*, *W*, and *C* are the height, width, and channel numbers of a feature map. It applies a single convolution operation conv() onto *X* followed by a sigmoid activation function σ that makes the weights belong to 0 to 1. Where *b* is the bias value of a convolution operator. Then it uses those weights $f_{weights}$ to re-weight the sum of the encoder features. Finally, the decoder feature *X* and the re-weighted features are concatenated $f_{output}$ as the input of next convolutional layer.

(1) $$f_{weights}=\sigma \left(conv\left(X\right)+b\right)$$

(2) $$f_{output}=concat\left(f_{weights}⊗\left(Y1⊕Y2\right),X\right)$$

where ⊗ and ⊕ denote the pixel-wise multiplication and sum operation, and concat(,) is a concatenate process on two features.

### 3.4. Loss Function

STAM is fed with a static image $x_{img}$ and its optical flow image $x_{of}$, and then a foreground mask $G(x_{img},x_{of})$ is generated. Manhattan distance is measured between the generated mask and ground truth one *y*. So the loss function of STAM is,

(3) $$L_{STAM}=\|G\left(x_{img},x_{of}\right)-y{\|}_{1}$$

STAM is trained by minimizing $L_{STAM}$. It detects foreground in each video frame by feeding spatio-temporal information without any post-processing like median-filtering.

### 3.5. Motion Cue

Sequences of ordered frames allow the estimation of motion as either instantaneous image velocities or discrete image displacements. Most of the pixel-level motion estimation method is based on optical flow. Optical flow field actually represents the motion vector of each pixel in the image, which are taken at times *t* and t+Δt at every pixel position, which can also be understood as the projection in a two-dimensional imaging plane of the movement field. There are a large number of an effective optical flow algorithm widely used in motion estimation tasks [41,42]. These methods are called differential since they are based on local Taylor series approximations of the image signal. They use partial derivatives with respect to the spatial and temporal coordinates. Optical flow indicates the global movement of the scene and local movement of objects where the moving areas have a high probability to carry foreground objects in real-world. So it provides prior knowledge to guide where it should be focused on in a scene. In this work, we employed Lucas and Kanade’s optical flow method [43] which makes use of the spatial intensity gradient of the images to find a good match using a type of Newton–Raphson iteration. This technique is fast because it examines few potential matches between the images.

### 3.6. Model Training

For all the scene-specific models, even each training set are with enough number of samples and achieve a high F-measure, this model could an over-fit specific scene and their generalization capabilities are limited. So we avoid training scene-specific models for every scene but use all of the scenes in CDnet 2014 to train a single model. Following the training setting in DeepBS [33], for the training data, we randomly select 5% samples with their ground truths of each subset from CDnet 2014. The left 95% samples are used to test the model. The optical flow of every video is extracted at a 50% down-sampling ratio in advance. All the frames, ground truths, and optical flow images are resized to 256×256. Since the optical flow of an image has only one channel, we extend the number of channels to 3 to match its original frame. The proposed model is trained for about 4.5 h in 100 epochs with 28 samples as a mini-batch. As for the optimizer, we use the Adam optimizer with $\beta_{1}=0.95$, $\beta_{2}=0.90$ and a small learning rate $3\times 10^{-5}$. The parameters in the proposed model are initialized randomly without any pre-trained model. The model is trained on two RTX2080TI GPU with Ubuntu 16.04 LTS OS and Tensorflow.

## 4. Experiments

### 4.1. Data Preparing and Experiment Setting

All the testing results on different scenes are given by the single STAM model. Segmented foreground was obtained without any post-processing.

In order to evaluate the proposed model, we employed the Change Detection 2014 dataset (CDnet 2014) [14], which contains various set of camera-captured videos including PTZ, bad weather (BDW), baseline (BSL), camera jitter (CJT), dynamic background (DBG), intermittent object motion (IOM), low frame rate (LFR), night videos (NVD), shadow (SHD), thermal (THM), and turbulence (TBL). The 95% samples in CDnet 2014 are used to test the model, without any overlap of the training set.

In order to test the foreground detecting in cross-scenes, Wallflower [44] and PETS [45] datasets were introduced. We applied STAM trained on CDnet 2014 to test these two datasets without any additional training phase.

In order to do the ablation experiments, we removed the attention module from STAM and concatenate the encoder feature and decoder feature straightly, called STAM${}_{NoAtt}$. We removed the encoder layers associated with optical flow from STAM, which output the foreground mask and thus relied only on static image, called STAM${}_{NoOF}$.

### 4.2. Results and Evaluation on CDnet 2014

**Figure 3:**
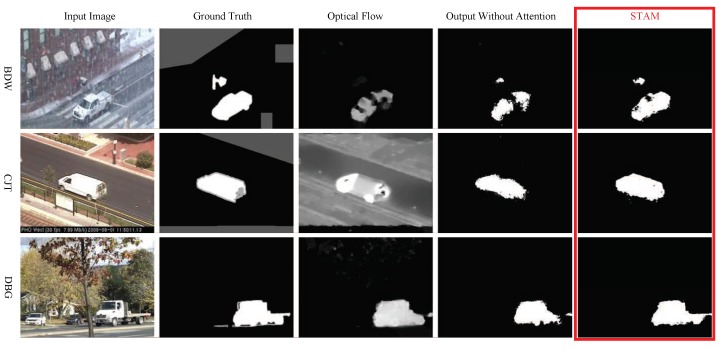
Comparison on the output samples of STAM and the model without attention model. Each column has five images and there are static image, ground truth, optical flow, segmented results of STAM${}_{NoAtt}$, and STAM, from left to right. Abbreviation: Bad weather (BDW), camera jitter (CJT), and dynamic background (DBG).

**Figure 4:**
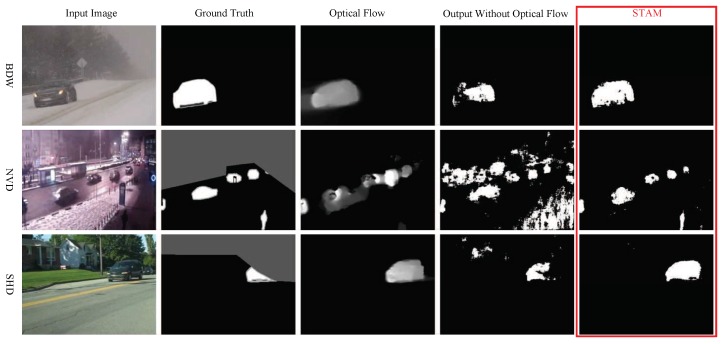
Comparison on the output samples of STAM and the model without optical flow. Each column has five images and there are static image, ground truth, optical flow, segmented results of STAM${}_{NoOF}$, and STAM, from left to right. Abbreviation: Bad weather (BDW), night videos (NVD), and shadow (SHD).

Figure 3 illustrates the samples segmented by the proposed model STAM and the model without attention processing. The STAM provides much clearer boundaries and accurate segmentation. As illustrated in Figure 4, the STAM took optical flow into consideration which helped to find objects hidden in static images and suppressed false alarm.

We computed seven different evaluation metrics for each algorithm compared in CDnet 2014, shown in Table 2. The STAM based method surpassed the state-of-the-art algorithms in most of the metrics. The Precision of STAM was 0.9851, while the Precision of Cascade CNN ranked second with 0.8997, and DeepBS ranked third with 0.8332. STAM improved the precision 9–15%. For Recall and FNR, CascadeCNN surpassed STAM, but by less than 1%. For F-measure, STAM outperformed by 4% than the rank second Cascade CNN. Meanwhile, the STAM containing the attention mechanism and temporal information exceeded models that excluded these parts, with F-measure increasing respectively by 9% and 6%.

**Figure 5:**
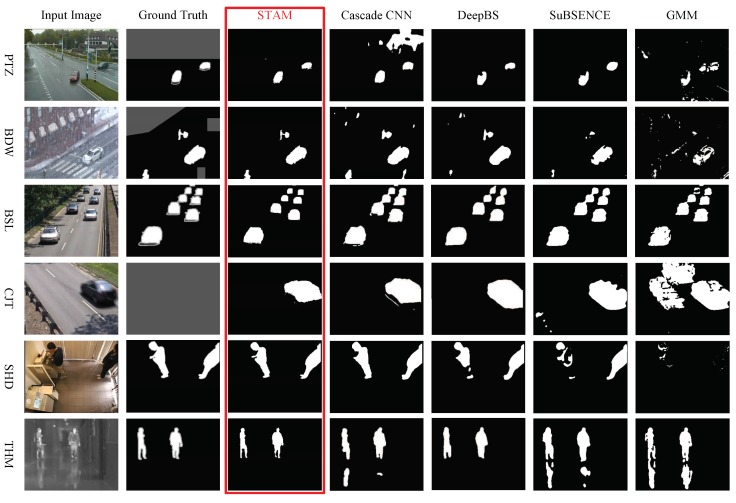
Samples segmented by different foreground detection methods among several scenes in CDnet-2014. Abbreviation: Bad weather (BDW), baseline (BSL), camera jitter (CJT), shadow (SHD), and thermal (THM).

**Figure 6:**
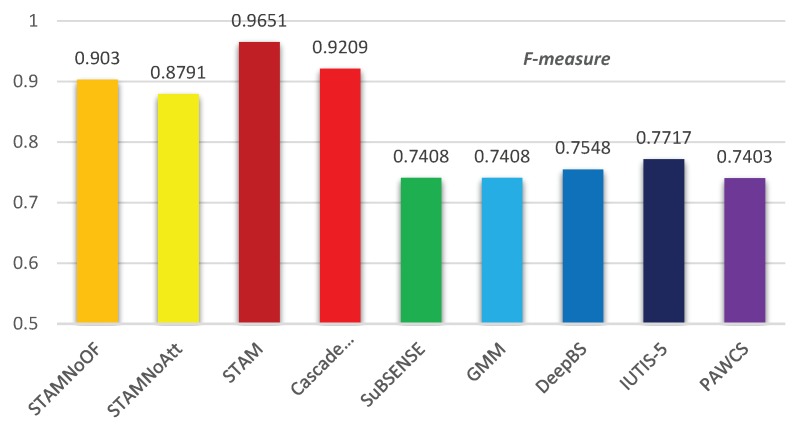
F-measure comparison. STAM outperformed by 4% over Cascade CNN which ranked second. Meanwhile, the STAM containing attention mechanism and optical flow exceeded models that excluded these parts, with a respectively increasing F-measure of 9% and 6%.

Table 3 represents the F-measures computed through STAM and the state-of-the-art approaches in different sub-sets. STAM gained the highest F-measure scores among the other algorithms in six out of 11 categories, under the cases of bad weather, intermittent object motion, shadow, thermal, turbulence, and light switch. The visualized results are provided in Figure 5 and the average F-measures of all the methods are illustrated in Figure 6.

Note that, STAM gave all the testing results on different scenes are given by this single model. While another model CascadeCNN was trained with a scene-specific style following its original experiment setting. For example, training a model on subset PTZ in CDnet2014 and tested on PTZ, while for another subset, Baseline, retrain the models and tested on Baseline. So their models could over-fit a specific scene. However STAM solved all the sub-scenes in CDnet2014 without retraining. However, the proposed model still brought improvement in scenes like bad weather, shadow, thermal, and overall performance. More importantly, compared to the state-of-the-art cross-scene single model DeepBS [33], the proposed model achieved significant improvements in all the seven metrics.

### 4.3. Cross-Scene Segmentation Results on Wallflower and PETS

We directly applied the STAM trained on CDnet 2014 to Wallflower and PETS without any tuning to test its capability to copy with cross scenes segmentation. There were seven different scenes in Wallflower, and only one hand-segmented ground truth was provided for each scene. Since there was no foreground illustrated in the ground truth of “Moved Object”, we excluded this scene in the experiments. Table 4 illustrates the quantitative results on Wallflower, showing that STAM presented a better performance on two subsets than DeepBS, and gained the best performance in overall F-measure. Quantitative comparisons on another dataset PETS are exhibited in Table 5.

**Figure 7:**
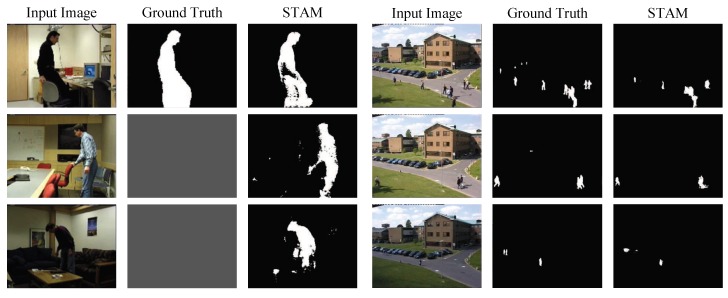
Foreground detection in Wallflower and PETS. The first three rows are from Wallflower while the other three rows are from PETS.

On PETS, we compared STAM with some background modeling approaches, including the newly proposed CPB+HoD approach [25]. The F-measure of STAM was comparable with the standard background modeling approach GMM without any training on PETS, but failed to outperform the CPB+HoD and Vibe approaches. The reason was that the proposed model emphasized a generalization performance via the process of big dataset training, but at the same time, it may have failed to preserve small details in a specific scene. In PETS dataset most of the foreground are quite small compared with the foreground in Wallflower dataset, so that its performance was not as good as in Wallflower dataset. Figure 7 illustrates some samples segmented by the proposed model on Wallflower and PETS datasets, which also indicates the weakness of STAM in preserving the detail of very small foreground.

The test speed of STAM is **10.8 fps** for the frame size 256 by 256 on a single GTX1080TI with a 32GB RAM and Ubuntu 16.04 LTS operating system.

## 5. Conclusions

We proposed a Spatio-Temporal Attention Model for cross-scene foreground detection. The benefit of the proposed model was that appearance and motion features, low-level, and high-level features are synthesized by attention modules via feature learning. The ablation experiments validated the model with an optical flow that had a 6% better F-measure than without it, Additionally, the model with attention had a 9% better F-measure than without it. The proposed model surpassed state-of-the-art methods under the cases of bad weather, intermittent object motion, shadow, thermal, turbulence, and light switch. It improve the overall precision by 9% and F-measure by 4% over a scene-specific model Cascade CNN. Quantitative and visualized performance on Wallflower and PETS benchmarks show its promising generalization ability of the scene without any additional training. Furthermore, it shows promise in processing surveillance videos in real time.

## Figures and Tables

**Table 1:** Filter size and output size of each layer in encoders, decoders, and attention modules.

Details	Encoder1	Encoder2	Encoder3	Encoder4	Encoder5	Encoder6	Encoder7	Encoder8
Filter	5 × 5	5 × 5	5 × 5	5 × 5	5 × 5	5 × 5	3 × 3	3 × 3
Output	128 × 128 × 64	64 × 64 × 128	32 × 32 × 256	16 × 16 × 512	8 × 8 × 512	4 × 4 × 512	2 × 2 × 512	1 × 1 × 1024
Details	Decoder1	Decoder2	Decoder3	Decoder4	Decoder5	Decoder6	Decoder7	Decoder8
Filter	3 × 3	3 × 3	5 × 5	5 × 5	5 × 5	5 × 5	5 × 5	5 × 5
Output	2 × 2 × 512	4 × 4 × 512	8 × 8 × 512	16 × 16 × 512	32 × 32 × 256	64 × 64 × 128	128 × 128 × 64	256 × 256 × 1
Details	Attention1	Attention2	Attention3	Attention4	Attention5	Attention6	Attention7	–
Filter	3 × 3	3 × 3	5 × 5	5 × 5	5 × 5	5 × 5	5 × 5	–
Output	2 × 2 × 1024	4 × 4 × 1024	8 × 8 × 1024	16 × 16 × 1024	32 × 32 × 512	64 × 64 × 256	128 × 128 × 128	–

**Table 2:** Average performance comparison of different methods over the 11 categories in CDnet 2014. Abbreviation: STAM${}_{NoOF}$ without optical flow and STAM${}_{NoAtt}$ without attention.

Method	Recall	Specificity	FPR	FNR	PWC	F-measure	Precision	Single/Scene-Specific
*STAM${}_{NoOF}$*	0.9294	0.9955	0.0045	0.0706	0.6682	0.9030	0.8781	**single model**
*STAM${}_{NoAtt}$*	0.8364	0.9977	0.0023	0.1636	0.7698	0.8791	0.9265	**single model**
*STAM*	0.9458	**0.9995**	**0.0005**	0.0542	**0.2293**	**0.9651**	**0.9851**	**single model**
Cascade CNN [34]	**0.9506**	0.9968	0.0032	**0.0494**	0.4052	0.9209	0.8997	scene-specific
SuBSENSE [46]	0.8124	0.9904	0.0096	0.1876	1.6780	0.7408	0.7509	scene-specific
GMM [18]	0.6846	0.9750	0.0250	0.3154	3.7667	0.5707	0.6025	scene-specific
DeepBS [33]	0.7545	0.9905	0.0095	0.2455	1.9920	0.7548	0.8332	**single model**
IUTIS-5 [47]	0.7849	0.9948	0.0052	0.2151	1.1986	0.7717	0.8087	scene-specific
PAWCS [48]	0.7718	0.9949	0.0051	0.2282	1.1992	0.7403	0.7857	scene-specific

**Table 3:** F-measures comparison of different methods on each category in CDnet 2014. Abbreviation: Bad weather (BDW), baseline (BSL), camera jitter (CJT), dynamic background (DBG), intermittent object motion (IOM), low frame rate (LFR), night videos (NVD), shadow (SHD), thermal (THM) and turbulence (TBL).

Method	PTZ	BDW	BSL	CJT	DBG	IOM	LFR	NVD	SHD	THM	TBL
*STAM*	0.8648	**0.9703**	**0.9885**	0.8989	0.9483	**0.9155**	0.6683	0.7102	**0.9663**	**0.9907**	**0.9328**
Cascade CNN [34]	**0.9168**	0.9431	0.9786	**0.9758**	**0.9658**	0.8505	**0.8370**	**0.8965**	0.9414	0.8958	0.9108
SuBSENSE [46]	0.3476	0.8619	0.9503	0.8152	0.8177	0.6569	0.6445	0.5599	0.8646	0.8171	0.7792
GMM [18]	0.1522	0.7380	0.8245	0.5969	0.6330	0.5207	0.5373	0.4097	0.7156	0.6621	0.4663
DeepBS [33]	0.3133	0.8301	0.9580	0.8990	0.8761	0.6098	0.6002	0.5835	0.9092	0.7583	0.8455
IUTIS-5 [47]	0.4282	0.8248	0.9567	0.8332	0.8902	0.7296	0.7743	0.5290	0.8766	0.8303	0.7836
PAWCS [48]	0.4615	0.8152	0.9397	0.8137	0.8938	0.7764	0.6588	0.4152	0.8710	0.8324	0.6450

**Table 4:** F-measures comparison of different methods on Wallflower dataset.

Category	*STAM*	DeepBS [33]	SuBSENSE [46]	PBAS [49]	GMM [18]
Bootstrap	0.7414	**0.7479**	0.4192	0.2857	0.5306
Camouflage	0.7369	**0.9857**	0.9535	0.8922	0.8307
ForegroundAperture	**0.8292**	0.6583	0.6635	0.6459	0.5778
LightSwitch	**0.9090**	0.6114	0.3201	0.2212	0.2296
TimeOfDay	0.3429	0.5494	0.7107	0.4875	**0.7203**
WavingTrees	0.5325	0.9546	0.9597	0.8421	**0.9767**
Overall	0.7138	**0.7512**	0.6711	0.5624	0.6443

**Table 5:** Recall, precision, and F-measure comparisons on PETS dataset.

Method	Recall	Precision	F-measure
*STAM*	0.7023	0.8514	0.7697
CPB+HoD [25]	0.7562	**0.9652**	**0.8480**
ViBe [1]	0.8821	0.7059	0.7842
GMM [18]	**0.9508**	0.6465	0.7697
KDE [2]	0.8836	0.5181	0.6531
